# New Approaches for Cancer Treatment: Antitumor Drugs Based on Gene-Targeted Nucleic Acids

**Published:** 2009-07

**Authors:** O.A. Patutina, N.L. Mironova, V.V. Vlassov, M.A. Zenkova

**Affiliations:** 1 Institute of Chemical Biology and Fundamental Medicine, Siberian Branch, Russian Academy of Sciences

## Abstract

Currently, the main way to fight cancer is still chemotherapy. This method of treatment is at the height of its capacity, so, setting aside the need for further improvements in traditional treatments for neoplasia, it is vital to develop now approaches toward treating malignant tumors. This paper reviews innovational experimental approaches to treating malignant malformations based on the use of gene-targeted drugs, such as antisense oligonucleotides (asON), small interfering RNA (siRNA), ribozymes, and DNAzymes, which can all inhibit oncogene expression. The target genes for these drugs are thoroughly characterized, and the main results from pre-clinical and first-step clinical trials of these drugs are presented. It is shown that the gene-targeted oligonucleotides show considerable variations in their effect on tumor tissue, depending on the target gene in question. The effects range from slowing and stopping the proliferation of tumor cells to suppressing their invasive capabilities. Despite their similarity, not all the antisense drugs targeting the same region of the mRNA of the target-gene were equally effective. The result is determined by the combination of the drug type used and the region of the target-gene mRNA that it complements.

## INTRODUCTION

At the current stage of modern medicine, one of the most important projects is to increase the effectiveness of cancer treatment by searching for and developing new therapies and improving traditional therapeutic approaches. A combination of surgery, radio- and chemotherapy is still the golden standard for cancer treatment, and these approaches have led to an 8-fold increase in patient survival over the last 30 years. The negative features of surgery-only treatment are recurrent tumors, the spread of metastases, and the formation of unresectable malignant malformations. This forces doctors to use radio- and chemotherapy. Alas, even this combination of powerful cancer therapies often doesn´t bring positive results. Therefore, despite the undeniable achievements of modern oncology, increasing the effectiveness of cancer treatment is of utmost importance.

During the last several decades, complex chemotherapy has become the main approach for treating cancer patients. Its use however is limited, despite the fact that it increases survival rates by 30% to 90%, depending on the type of malformation. The main hindrances are systemic toxicity, nonselective action (the effect is not specifically targeted towards tumor tissue), and the emergence of drug-resistant tumor cell clones.

Recent discoveries have provided scientists with detailed knowledge of the molecular processes underlying carcinogenesis, tumor invasiveness, angiogenesis, and metastasis, as well as other processes, such as tumor suppression, growth control, apoptosis, and immune response. These data have led to the development of a new generation of chemotherapeutic drugs, such as Gleevec (aka Glivec or Imatinib mesylate), Mabthera (aka Rituximab), etc., which have a highly selective effect on their cellular target. It is well known that creating a new drug takes about 10-20 years of research, and improving its selectiveness increases its cost manifold. Currently, chemotherapy as a high-dose active attack aimed at tumor cells is at the limit of its ability. Despite the achieved level of patient survival (for certain cancer types it has made a 10-fold increase in the last 20 years), there is still a 10% to 70% proportion of patients who, for a number of reasons, do not react to treatment. Therefore, the creation of new methods of therapy is a relevant problem at this time. Among the drugs which are currently being developed, gene-targeted drugs are of considerable interest. The possibility of inhibiting a gene´s expression was first discovered in the ground-breaking research of N.I. Grineva and her colleages [1-3] and was studied further in order to regulate the expression of genes involved in carcinogenesis using antisense [4] and gene-targeted oligonucleotides [5]. Currently, the main lines of inquiry into gene-targeted cancer therapy are strategies to suppress oncogene overexpression, restore the expression of tumor suppressing genes, boost the activity of the immune system, suppress angiogenesis and metastasis, and initiate tumor self-destruction.

This paper reviews the new experimental approaches to cancer treatment based on gene-targeted oligonucleotides, which are currently being used in experiments on cell cultures and laboratory animals. Some of these drugs are also in various stages of clinical trial.

## How Gene-Targeted Nucleic Acids Work

Antitumor drugs based on nucleic acids are highly specific tools which allow the gene expression to be regulated, and they have been attracting the attention of scientists as possible regulators of carcinogenesis at the molecular level. The suppression of several genes whose anomalously high expression is associated with neoplastic transformation can be achieved by nucleic acid-based drugs, such as antisense oligonucleotides (asON), small interfering RNAs (siRNA), ribozymes, and DNAzymes. Generally speaking, the mechanism of gene suppression by these drugs is the complementary binding of oligonucleotides to their mRNA target, which causes the target mRNA molecule to be destroyed or blocks its translation.

**Fig. 1.**
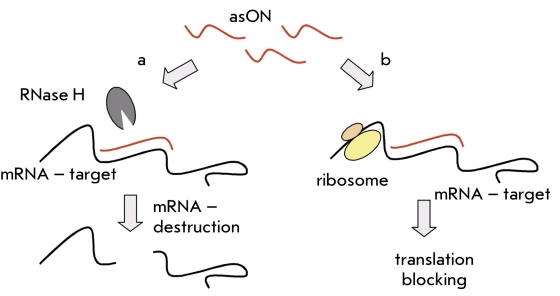
How antisense oligonucleotides (asON) work. (a) RNA is cleaved as a part of a heteroduplex with the asON by Rnase H. (b) Blockage of translation caused by binding of the oligonucleotide onto the mRNA

AsON are synthetic single-strand DNA, 15-20 nucleotides long, and they can form a complementary complex with the target mRNA sequence [6]. Protein synthesis is suppressed by asON due to the fact that the mRNA target is degraded by the intracellular RNase H, which identifies the hybrid DNA/RNA complex (Fig. 1a), or due to a block of translation, since the formation of a hybrid complex hampers the ribosome´s movement on the mRNA strand (Fig. 1b) [7]. Recently discovered asON can block the transfer of spliced mRNA from the nucleus to the cytoplasm; other asON can block a splicing site in pre-mRNA and thus cause the expression of an alternate protein product. [8, 9].

The ability of asON to selectively suppress the production of a protein was demonstrated by Zamechnik and Stephenson in 1978 [4]. They showed that a 13-base oligonucleotide complementary to the 3'-terminal sequence of the RNA of the Rouse sarcoma virus inhibits viral replication in vitro. This study led to the research of asON as a potential therapeutic method in cancer and viral infection treatment, as well as treatments for inflammatory processes, blood diseases, and cardio-vascular conditions [10-14].

Since it is known that naturally occurring oligodeoxyribonucleotides are rapidly degraded by nucleases in vivo and in cell cultures, several chemical modifications are incorporated into the asON structure to increase their stability [11]. These modifications increase not only asON stability against nucleases, but also their biological effectiveness, hybridization efficiency, and cellular uptake. Among the most notable antisense oligonucleotide derivates are thiophosphate oligonucleotides, in which one of the oxygen atoms not incorporated in the phosphodiester bond is substituted for a sulphur atom [11, 15]. Thiophosphate asON are resistant to nucleases, highly soluble, effective in hydridization, and form a heteroduplex with mRNA, which is targeted by RNase H [11]. One drawback of thiophosphate asON is their high affinity to a range of proteins [16, 17]. Second-generation asON carried an alkyl residue in the ribose 2´-position: 2´-O-methyl and 2´-O-methoxyethyl oligoribonucleotides were effective in blocking mRNA translation, but they did not activate the degradation of the mRNA/asON heteroduplex by RNase H. [18]. Later Nielsen and co-authors substituted the sugar-phosphate backbone of the nucleic acid to an N-(2´-aminoethyl)-glycine polyamide structure [19], which gave rise to polyamide nucleic acids (PNA). PNA are biologically stable and hybridize effectively, although they do not activate RNase H. Also, PNA are neutral molecules, which poses difficulties for solubilization and cellular uptake [20, 21]. Aside from PNA, third-generation asON are N3´-N5´-phosphoramidates (NP), in which the 2´-deoxyribose 3´-hydroxyl group is substituted for a 3´-aminogrop [22], and morpholino-oligonucleotides (MF), whose backbone is based on morpholine and a dimethylamidephosphate linker [23]. On the molecular level, these oligonucleotides block translation by way of the asON binding to the target mRNA and/or by modulating splicing. [23]. NP and MF oligonucleotides are used primarily for developmental biology studies on zebra fish (Danio rerio) embryos [24].

Some of the more promising chemically modified oligonucleotides are LNAs (Locked Nucleic Acids). These are oligonucleotides with an additional structural element, a 2´-O,4´-C-methylene linker, which fixes the sugar residue in the C3´-endo-conformation [25, 26]. LNA are resistant to degradation by nucleases and have a very high affinity towards nucleic acids. The promise of LNA use in vivo is supported by their extremely low toxicity when injected intravenously or microinjected directly into the brains of animals. [27].

**Fig. 2.**
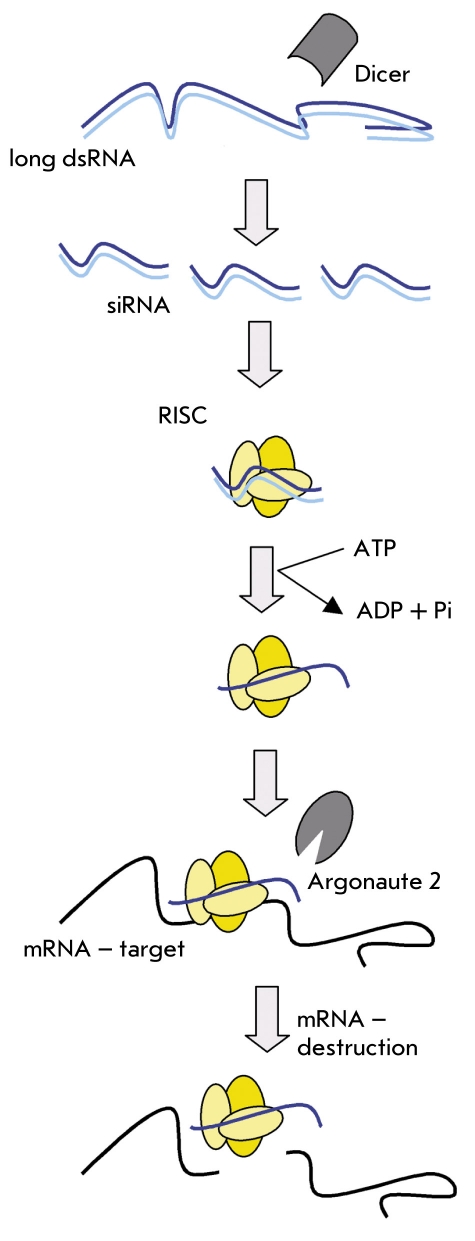
How RNA interference happens

RNA interference (RNAi) was first discovered on the nematode Caenorhabditis elegans (C. elegans) as a biological response to the introduction of foreign double-stranded RNA (dsRNA). This response brought on the specific suppression of the respective genes´ expression (gene silencing) [28]. RNAi is an evolutionarily conserved mechanism which allows the organism to defend itself against an invasion of foreign RNA such as viruses [29, 30]. After entering the cell, exogenous dsRNA is processed into small interfering RNA (siRNA) by an intracellular ribonuclease called Dicer [31]. These siRNAs are about 21-22 nucleotides long and are incorporated into the multiprotein RISC complex (RNA-induced silencing complex). siRNA that are part of the RISC complex specifically bind to the complementary mRNA sequence, which is then degraded by the ribonuclease Argonaute 2, which is also a member of the RISC complex (Fig. 2) [30, 32]. After that, the siRNA molecules are used repeatedly to destroy more molecules of the complementary mRNA, which leads to very efficient gene silencing [32]. The specific abrogation of gene expression can be achieved by using synthetic siRNA, or siRNA enzymatically constructed in vitro, as well as by using short hairpin RNA (shRNA), which are expressed in the cell from DNA templates obtained by PCR or included into DNA vectors [33].

**Fig. 3.**
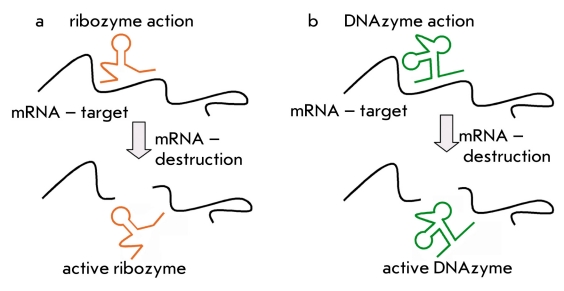
Processes of ribozyme action (a) and DNAzyme action (b)

During the 1980s, catalytic RNA molecules were discovered. These molecules could cleave RNA and are called ribozymes [34]. Naturally occurring catalytic RNA are divided into large and small ribozymes. Large ribozymes include RNA which is encoded by introns of groups I and II and also the RNA subunits of RNase P. Small ribozymes are hammer-head-like ribozymes, hairpin ribozymes, hepatitis D virus ribozymes, and Varkud Satellite RNA ribozymes [35, 36]. RNA degradation by ribozymes is a 3-step process. First the ribozyme binds to a complementary sequence by forming classical Watson-Crick base pairs; then it cleaves the RNA substrate at a specific site; and, finally, it releases the degradation products (Fig. 3a) [36].

Almost all the ribozyme types are being tested as therapeutic drugs, but the hammer-head-like ribozymes are being used more, because they have been studied more extensively [35]. This ribozyme cleaves the target RNA primarily at the NU H triplet (N is any nucleotide and H is any nucleotide except guanosine), with AUC and GUC sequences being the most effective processing sites [37]. Another ribozyme often used in therapeutic studies is the hairpin ribozyme [38]. The hairpin ribozyme cleaves the target RNA at the N*GUC sequence (N is any nucleotide).

DNA molecules which exhibit catalytic activity have yet to be discovered in nature. In 1997 Santoro and Joyce, using the SELEX in vitro selection procedure, obtained oligodeoxyribnucleotides that could catalyze RNA cleavage. These molecules were named deoxyribozymes or «10-23» DNAzymes [39]. «10-23» DNAzymes are single-stranded DNA molecules which have a conserved catalytic core of 15 nucleotides flanked by two variable oligonucleotide sequences. These flanking sequences facilitate the formation of a complementary complex with the target RNA. (Fig. 3b) [39]. RNA molecules are cleaved between the unpaired purine and the paired pyrimidine in the presence of magnesium ions. The most effective cleavage is achieved at the AU and GU sites.

## Target Genes for Drugs Based on Gene-Targeted Nucleic Acids

A key role in carcinogenesis is played by the change in the expression levels of certain genes whose anomalous expression leads to the defective regulation of cell proliferation, apoptosis, differentiation, and invasion. [40]. At the molecular level, malignant transformation is a complicated cascade of reactions; therefore, the effects of oncogenes are often multifunctional and tightly interconnected [40]. Since oncogenes are transcription factors and components of the signal transduction machinery of the cell, they are involved in many regulatory pathways, such as cell proliferation, the inhibition of apoptosis, invasion, etc. The main target genes for gene-targeted therapy are listed in Table 1.

**Table 1:** A list of target genes for drugs based on gene-targeted nucleic acids

Carcinogenic events	Target gene	Function	Drugs used to suppress function
Proliferation	Ras oncogenes (*K-ras*, *H-ras* and *N-ras*)	Part of the cellular signal transduction system and regulates a wide range of processes, such as proliferation, differentiation, and survival [42]	asON, ribozymes, siRNA
	*c-myc*	Activates the proliferation of tumor cells (regulates the cell cycle and telomerase activity) [49, 160]	asON, siRNA, DNAzymes
	*PKC-α*	Is involved in the cellular signal transduction and controls proliferation and cell survival [53, 54]	asON, ribozymes, DNAzymes
	*Clusterin*	Is involved in lipid transport, cell division, and apoptosis; supports cell survival in response to therapy; and increases tumor drug resistance [57, 58]	asON
	*IGF-1R*	Activates the MAPK and PI-3K signal pathways, which stimulate proliferation and mitogenesis and inhibit apoptosis [61–63]	asON
Blocking of apoptosis	*bcl-2*	Negativly regulates apoptosis by blocking the excretion of cytochrome c from the mitochondria into the cytoplasm [67]	asON, siRNA, ribozymes
	*Survivin*	Regulates cell division (interacts with microtubules in the mitotic spindle and promotes mitotic entry through G2/М checkpoint) and suppresses apoptosis (inhibits the inner caspase-9-dependant apoptosis pathway) [73, 74]	asON, ribozymes
	*bcr-abl*	Stimulates a mitogenic and antiapoptotic signal mediated by Ras-regulated signal pathways [81, 82]	Ribozymes, DNAzymes, siRNA
	*с-raf*	Activates the MAPK/ERK cascade and negative regulation of apoptosis by inactivating the proapoptotic Bad protein [88, 90]	asON
Drug resistance	*MDR1*	Forms transmemrane channels for ATP-dependant expulsion of drugs out of the cell, which endows tumors with drug resistance [95–97]	asON, siRNA, ribozymes
	*γ* – glutamylcysteine syntetase (glutathione system)	Intracellular detoxication of anticancer drugs [95]	Ribozymes
Dysfunction of tumor suppressor genes	*DNMT1*	Hypermethylation and inactivation of tumor suppressor genes [102, 103]	asON
Increase of tumor cell lifespan	*hTERT*	Hyperactivation of the telomere repeat elongation machinery and, as a result, an increase in the malignancy and the lifespan of transformed cells [109]	asON, ribozymes
DNA synthesis arrest	*RRR2* or *RRM2*	Controls the amount of deoxyribonucleotides needed for DNA synthesis via regulating the conversion of ribonucleotides into deoxy ribonucleotides [113]	asON, siRNA
Tumor angiogenesis	*Flt-1* (VEGFR)	Active neovascularization and suppression of the anti-tumor immune response [119, 120]	asON, ribozymes, DNAzymes, siRNA
	*neu* (HER2 or ErbB2, EGFR family)	Activation of signal transduction pathways, which lead to the stimulation of tumor progression events such as proliferation, invasion, and apoptosis inhibition [127,128]	asON, ribozymes
	*eIF4E*	Increases the translation of growth factors such as VEGF, С-myc, surviving, etc. [133 - 135]	asON
	*PTN*	IGrowth factor, promotes active tumor growth and vascularization [137 - 139]	Ribozymes
	*ALK*	Is a tyrosine kinase PTN receptor, facilitates its function, thus promoting tumor vascularization [141]	Ribozymes
Tumor invasion	*MMP9*	Elimination of components of the extracellular matrix and basal membrane and promotes tumor invasion [144 – 146]	Ribozymes, siRNA
	*FGF-BP*	Activation of FGF-2, which induces tumor cell proliferation and increases the invasive and angiogenic potential [148]	Ribozymes
	*Egr-1*	Activates proliferation and tumor invasion and is involved in establishment of the MDR phenotype [154,155]	DNAzymes
	*FAK*	Regulates adhesion and invasion into the extracellular matrix [158]	siRNA
	*CXCR4*	Stimulates metastatic processes [159]	siRNA

Firstly, malignant cell growth is based on the autonomous and unlimited proliferation of the cell clone. That is why researchers are most interested in genes which control proliferation and cell cycle progression such as c-myc, ras, genes encoding PKC-α (protein kinase C-α) and IGF-1R (insulin growth factor-1 receptor). Another effective approach would be to target the programmed cell death system by inhibiting the expression of antiapoptotic genes such as bcl-2, survivin, etc.

Proteins of the Ras family (K-ras, H-ras, and N-ras) are among the best studied molecules involved in transducing signals from tyrosine kinase receptors to the nucleus [41]. Overexpression or a point mutation of the ras gene found in certain types of oncological diseases causes the Ras protein to lose its ability to dephosphorylate; therefore, it stays constantly activated, imitating and transducing signals that stimulate proliferation and promote tumor-cell survival [42]. Genes of the Ras family are good targets for gene-targeted inhibition therapy. Notably, ribozymes have a characteristic way of inhibiting the activity of the ras oncogene which involves an increase in the degree of tumor cells´ differentiation [43-47]. But the leader among the gene-targeted drugs which suppress ras oncogene expression is asON ISIS 2503. The results of phase II clinical trials for the combined use of asON ISIS 2503 and gemcitabine were announced in 2004 [48].

The important role of the c-myc oncogene in cell proliferation and malignant transformation was discovered in the late 1970s by Bishop [49], and this protein was among the first that were tested as a target for antisense therapy. The premier drug which regulates c-myc expression is a morpholine oligonucleotide asON AVI-4126, which blocks the production of the protein by steric inhibition of translation, as opposed to RNase H activation [50]. This drug has successfully passed preclinical trials and has been shown to be well tolerated by patients. It is now in the second phase of clinical trials [51, 52].

The protein kinase C gene family is a group of serinethreonine kinases which are involved in regulating vital cellular functions such as differentiation, cell-cell interactions, secretion, cytoskeleton functions, gene transcription, proliferation, and apoptosis [53]. Among a dozen of isoforms of this protein, only PKC-α is shown to be involved in cell survival, proliferation and apoptosis [54]; it is also actively involved in neoplastic transformation of the cell. The best candidate for selective suppression of the tumor-specific PKC-α isoform turned out to be the thiophosphate asON ISIS 3521 (AffinitakTM, USA), which selectively binds the PKC-α mRNA and does not interact with the other, non-oncogenic members of the PKC family [55]. This drug has been approved for clinical trials.

Clusterin was first described in 1983 as a secretory glycoprotein [56] associated with a wide variety of physiological and pathological processes, such as lipid transport, tissue transformation, cell membrane defense, apoptosis, and the complement system function [57]. Later research showed that clusterin is a chaperone-like protein which increases cell survival in response to stress [58]. The inactivation of this gene by gene-directed drugs could compromise the DNA repair-system response to external hazards, such as chemotherapy. This turned out to be true; for instance, asON OXG-011 increases the toxic effect of paclitaxel on tumor cells in mice [59] and is on the clinical path in combination with chemotherapeutic drugs [60].

IGF-1R (insulin growth factor-1 receptor) is a transmembrane protein kinase which promotes independent cell growth [61]. Anomaly in IGF-1R expression is well known to be connected with carcinogenesis [61]. It was demonstrated that IGF-1R overexpression promotes the development of P-glycoprotein- and Bcl-2-mediated multiple drug resistance (MDR) [61, 63]. Suppressing the IGF-1R gene with a gene-targeted antisense oligonucleotide prevents tumor formation in mice in ex vivo1 experiments [64], which confirms the idea that asON are promising drugs to counteract the cancer cell defense system.

Here and further in the text, an ex vivo experiment is defined as tumor material being removed from the organism, treated by a gene-targeting drug in vitro, and introduced back into the organism.

The Bcl-2 protein is the major representative of a family of pro- (Bax, Bak, Bad) and anti- (Bcl-xl, Mcl-1) apoptotic factors and was first discovered in B-cell lymphoma cells in 1985 [65]. Bcl-2 gene expression is associated with aggressive tumor behavior in response to chemo- and radiotherapy [66]. Excess Bcl-2 protein promotes the inhibition of mitochondrial membrane depolymerization, thus blocking apoptotic mechanisms which are triggered by the therapy [67]. Suppressing bcl-2 expression with gene-targeted oligonucleotides facilitated apoptosis induction in tumor cells and increased cell sensitivity to apoptosis-inducing chemotherapeutic drugs [68, 69]. AsON G3139 showed a considerable therapeutic effect and is currently in phase III clinical trials [70-72].

Survivin is a member of the apoptosis-inhibiting protein family (IAP). Despite the fact that its precise function in the cell is yet unclear, it was shown that this protein is involved in regulating cell division and apoptosis [73]. Survivin selectively inhibits the inner caspase-9-dependant apoptotic pathway [74], and it also bypasses apoptosis mechanisms by interacting with the microtubules of the mitotic spindle, which promotes mitotic entry through the G2/M checkpoint [75] and in turn stimulates anomalous cell cycle progression. Suppressing the survivin encoding gene with asON led straight to apoptosis induction and tumor cell death [76, 77]. The inhibitory effect of a ribozyme on the activity of the gene in the absence of additional apoptosis inducers did not affect the viability of cells [78-80]. There have been no in vivo studies of survivin as a target gene so far.

The chromosome translocation t(9;22) combines two independent genes - bcr, which is located on the human chromosome 22, and abl, located on chromosome 9 - and thus forms a hybrid oncogene [81]. Like the original abl gene, the chimeric bcr-abl gene shows increased kinase activity. By phosphorylating certain cell factors, BCR -ABL facilitates malignant transformation and blocks apoptosis [82]. Specifically, the appearance of bcr-abl can lead to myeloid or lymphoid leukemia [83]. The hybrid nature of this protein presented several difficulties in trying to affect its production. In some cases, the gene-targeting drugs suppressed not only the chimeric gene expression, but also that of the original abl [84]. It turned out that Maxizyme, a double ribozyme which could cleave the target mRNA at two sites, was an effective tool for circumventing this non-specificity [85]. The drug Imatinib, which specifically inhibits tyrosine kinase, was developed for preventing chronic myeloleukemia and can be used to effectively control leukemia progression [86]. However, there are known cases of the disease where the tumor is resistant to Imatinib because of point mutations in the gene or for other reasons. In such cases, RNAi methods can be helpful. Experiments using siRNA have shown that BCR -ABL+ cells can be sensitized to Imanitib by RNAi [87].

Proteins of the Raf family are serine-threonine kinases which transduce signals from a wide variety of membrane growth factor receptors to apoptosis regulators. It was determined that the functionally active Raf-1 kinase activates the MAPK/ER K pathway (mitogen-activated protein kinases/extracellular signal-regulated kinases) [88], takes part in the signaling pathways of proliferation and cell survival via NF-κB [89], and inhibits the proapoptotic Bad protein [90]. Thus, Raf-1 is at the center of a network of vital signalling pathways; thus, mutations or defective expression of the raf-1 gene play a considerable carcinogenic role in cell transformation. In addition, Raf-1 is an effector of the protein product of the ras oncogene [91], which is often found to be mutated in malignantly transformed cells; this is why therapy directed at suppressing raf-1 can prove to be effective against ras-mediated neoplasias. The most promising results of specific c-raf gene suppression and the corresponding antitumor effect were obtained using asON. Investigators reported the results of phase II clinical trials in 2002 using a drug based on a thiophosphate asON [92-94].

In general, about 30% of the patients who receive traditional chemotherapy develop multi-drug resistance related to the MDR1 gene [95-97], which encodes the P-glycoprotein. P-glycoprotein is a member of the ABC-transporter protein superfamily, which uses ATP-hydrolysis to expel chemotherapeutic drugs from the cell. P-glycoprotein hyperactivity endows tumor cells with resistance to a wide variety of chemotherapeutic drugs. Because of this, inactivating the MDR1 gene can facilitate chemotherapeutic drug retention in cells and cause their death. MDR1 is a fairly common target for gene-targeted oligonucleotide drugs. Among these, ribozymes and siRNA are currently considered the most effective. These drugs almost entirely blocked tumor growth in mice [98, 99].

One of the most important cytostatic drug neutralizing systems of the cell is the glutathione system. Glutathione is a nonprotein thiol which has a sulfhydryl group that can interact with the reactive group of a drug and, as a result, form a conjugate of glutamine with the drug [95]. These conjugates are less reactive, more soluble, and are expelled from the cell by transporter proteins [95]. Thus, the activation of the glutathione system genes can cause tumor drug resistance [100]. To circumvent MDR caused by the glutathione system, a ribozyme that effectively restored tumor cell sensitivity to chemotherapeutic drugs was constructed [101].

Many studies have shown anomalous methylation at certain sites in the genome in tumor cells [102]. The enzyme DNMT1 (DNA methyltransferase 1) catalyses the transfer of a methyl residue from S-adenosinemethionine to the 5th position of the cytosine residues in CpG islets, affecting the expression of certain genes [103]. It was shown both that tumor cells have elevated DNA methyltransferase activity [104] and that the initiated hyperactivity of this enzyme leads to malignant transformation [105]. Furthermore, it is alleged that anomalies in methylation processes are a key factor in determining the tumors´ response to chemotherapy [106]. Antisense inhibition of DNMT1 gene expression led to the restoration of the function of tumor suppressor genes and increased tumor cell death [107]. The artificial antisense oligonucleotide MG98 is currently in phase II clinical trials [108].

The telomerase reverse transcriptase restores the telomere length by adding tandem repeats (TT AGGG) and is needed to fully replicate the ends of chromosomes [109]. It was demonstrated that the hyperactivation of hTERT (human telomerase reverse transcriptase) and carcinogenesis are highly correlated [110]. In order to suppress the gene´s activity, several differently modified asON were constructed, including a 2´-O-methyl asON, PNA [111] and a 2´5´-oligoadenilate oligonucleotide [112]. The 2´-O-methyl asON suppressed hTERT expression in a cell culture by 97% [111], and the 2´5´-oligoadenilate oligonucleotide caused a 50% regression of tumors [112].

Ribonucleotidereductase catalyzes the synthesis of 2´-deoxyribonucleotides from the corresponding ribonucleoside 5´-diphosphates. This is the limiting step in the formation of 2´-deoxyribonucleoside-5´-triphosphates, precursors in DNA synthesis [113]. The R2 subunit of ribonucleotidereductase (RRR 2) is synthesized in the late G1 phase and the early S phase and is a key factor in determining the rate of DNA replication [113]. Also, it is well known that RRR 2 plays a significant role in determining the malignant potential of cells by acting synergistically with certain oncogenes and being connected with the membrane protein Raf-1 and the mitogen-associated protein kinase 2 (MAPK2) [114, 115]. For this reason, the specific suppression of the RRR2 mRNA can cause a whole array of antineoplastic effects via a wide variety of mechanisms. AsON GTI-2040, which suppressed RRR2 expression, caused a 98% regression of renal carcinoma transplanted into mice [116]. Clinical trials showed that this drug was well-tolerated by patients [117].

In the early 70s Folkman proposed that the growth of solid tumors and metastasis are critically dependant on angiogenesis - the formation of new blood vessels from the surrounding vascular network [118]. The pathological growth of new vessels promotes solid tumor progression and metastasis. Over the last few decades, the main mediators of angiogenesis have been identified and characterized, providing researchers with novel targets for cancer therapy. Among the numerous neoangiogenic factors, the most prominent is VEGF (vascular endothelial growth factor) [119]. By activating receptors VEGFR-1 and VEGFR-2, VEGF induces the activity of intracellular signaling tyrosine kinases, which play a central role in stimulating the proliferation of endothelium cells [120]. At least 5 VEGF isoforms are generated by alternative splicing: VEGF206, VEGF189, VEGF165, VEGF145, VEGF121 [121, 122]. It has been shown that increased angiogenesis and tumor progression are associated with the overexpression of isoform VEGF165 [123]. Specific oligonucleotides and siRNA were developed in order to suppress the expression of the VEGF-encoding gene [124, 125]. An interesting approach towards inhibiting VEGF activity was the construction of ribozymes, and later DNAzymes, targeted at inhibiting the expression of Flt-1, which encodes the VEGF receptor VEGFR-1, and the KDR gene, encoding VEGFR-2 [126]. Among all the proposed strategies of VEGF inhibition, this approach proved to be the most effective, and the ribozyme-based drug Angiozyme, targeting the Flt- 1 gene, is currently in phase II clinical trial [35].

The protooncogene neu, also known as HER-2/erbB-2 or NGL, encodes a transmembrane receptor that exhibits tyrosine kinase activity, which is important for intracellular signal transduction [127]. Normally, the HER -2 protein is not produced in most human tissues, and in neoplasias this receptor exhibits tyrosine kinase activity even when there is no ligand to activate it [128]. The overexpression of HER - 2 in carcinogenesis is often attributed to the amplification of the corresponding gene [129]. The recently developed drugs Herceptin and Rituxanar are based on monoclonal antibodies that bind the HER -2 receptor, but clinical trials showed some negative side-effects, the worst being cardiotoxicity [130, 131]. This is why the gene-targeted therapy of HER -2-mediated neoplasias looks so promising. Of all the drugs based on nucleic acids and used to suppress HER -2 activity, the drug Herzyme, based on a hammer head ribozyme, is currently in clinical trials. Phase I clinical trials showed that the drug is well tolerated by patients [132].

The eukaryotic translation initiation factor 4E (eIF4E) binds to the capped 5´ terminus of cellular mRNAs and delivers them into the eIF4E translation initiation complex. This complex reads the mRNA sequence in the 5´-3´ direction and unwinds the secondary mRNA structure in the 5´-untranslated region, thus uncovering the translation start codon and promoting ribosome binding [133, 134]. In normal conditions, eIF4E activity is down-regulated by a specific eIF4E- binding protein 4E-BP (4E-binding protein). Malignant transformation is often accompanied by eIF4E overexpression or by the phosphorylation of 4E-BP. This leads to the release of an active eIF4E which forms the eIF4E translation complex [135]. Notably, many researchers have shown that hyperactive eIF4E in tumors mostly enhances the translation of proteins which are involved in tumor progression such as Bcl-2, survivin, cyclin D1, C-myc, and VEGF [133, 134]. Because of this, the specific suppression of eIF4E activity can turn out to be very useful in inhibiting tumor progression. An asON complementary to eIF4E showed good results. When injected into tumor-bearing mice, it caused a 10-fold regression of the tumor and had no noticeable side effects [136].

Pleiotropin (PTN ) is a secreted growth factor which is produced in large quantities during the development of the nervous system and «turned off» in adults [137], except in some cancer patients [138]. PTN is an active mitogen for fibroblasts and epithelial cells [137, 138]. Also, it can induce the release of active proteolytic enzymes from endothelial cells [139]. These data point to the potentially crucial role of PTN in angiogenesis. In order to suppress the expression of PTN in tumor cells, a ribozyme complementary to the PTN mRNA was constructed. It showed considerable antitumor and antimetastatic activity [140]. Moreover, it was recently discovered that the inhibition of the expression of anaplastic lymphoma kinase (APK), which causes the development of anaplastic lymphoma [141] and functions as a pleiotropin receptor [142], did not only cause tumor regression, but also caused a twofold increase in mean survival time in mice [143].

Tumor progression is accompanied by the tumor´s ability to spread beyond the boundary of its own tissue and to continue to grow into nonrelated tissues. Modulating the expression of genes which are involved in stimulating migration and invasion by means of gene-targeted drugs is a strategy that is often used by researchers. Matrix metalloproteinase- 9 (MMP9) can initiate the degradation of certain components of the extracellular matrix and the basal membrane (collagens IV and V, elastin, entactin, casein, and galectin) [144, 145], which promotes the epithelial-mesenchymal transition of tumor cells and stimulates metastasis [146]. The suppression of metalloproteinases by ribozymes partially abrogates metastasis and increases the mean survival time of tumor-bearing mice, but it does not cause tumor regression [147].

The fibroblast growth factor (FGF) is interesting to researchers because it is a powerful mitogen which induces differentiation and angiogenesis during development and also stimulates tumor cell invasion [148]. Normally, adults produce only a small quantity of FGF, but during certain oncological diseases, FGF production is elevated [149]. Secreted FGF binds tightly to heparan sulphate proteoglycans in the extracellular matrix, thus blocking FGF activity [150]. One of the mechanisms of FGF release is binding to FGFBP (FGF-binding protein), which mobilizes and activates FGF [150]. Studies have shown that FGF-BP is expressed in certain carcinomas [151] and also promotes the conversion of nononcogenic FGF-expressing cell line SW-13 to oncogenic and angiogenic phynotype [150]. A ribozyme designed to suppress the FGF-BP gene expression effectively abrogated tumors in mice [152].

The early growth response factor-1 (EGR-1) is a typical representative of a family of transcription factors which possess a «zinc-finger» structural domain [153]. EGR-1 activity is induced by a number of external and intracellular stimuli such as growth factors, cytokines, ultraviolet light, ionizing radiation, etc [153]. It has been shown that EGR-1 is involved in multiple regulatory pathways in tumor cells. Its activity is involved in the development of malignant transformation [154], in the regulation of MDR1 gene transcription [155], and in the negative response to estrogen in mammary gland carcinoma [156]. In order to suppress EGR- 1 gene expression, a DNAzyme, which caused a 3-fold decrease in tumor size in mice, was designed [157].

The focal adhesion kinase (FAK) is a non-receptor tyrosine kinase, located in integrin clusters, through which the cytoskeleton interacts with proteins of the extracellular matrix (focal adhesion sites). FAK receives signals from growth factors and adhesion factors and transmits them into the cell. FAK is an important mediator of signal transduction pathways, regulating proliferation, migration and cell viability and it is often overexpressed in tumor cells. siRNA effectively suppresses FAK activity and tumor growth in mice [158].

In mammary gland tumors, the transformed cells express the chemokine receptor CXC (CXCR 4), which causes metastasis into organs containing a lot of CXCR 4 ligands. The inhibition of CXCR 4 expression with siRNA suppresses the adhesive and invasive properties of tumor cells [159].

## Application Gene-Targeted Nucleic Acid Drugs in Tissue Cultures , Experimental Animal Models and Clinics

Drugs based on nucleic acids have attracted the attention of researchers for a long time, being potentially useful for gene-targeted cancer therapy due to their ability to interact with the key pathways of carcinogenesis. Table 2 sums up the main results of in vitro studies in this area and Table 3 combines the data of preclinic in vivo tests and clinical trials. In this section, we describe the development of drugs based on gene-targeted oligonucleotides which specifically inhibit the functions of target genes involved in carcinogenesis.

**Table 2:** Results of nucleic acid-based drugs testing in vitro

Target-genes	Drug	Tumor type	Effect
*H-ras/K-ras*	asON	Carcinoma of the uteral cervix [161], hepatoma [162]	A decrease in H-ras-luciferase-mRNA level by 98% [161]; inhibition of cellular growth by 87.8%, block of the entry into the S-pahse of the cell cycle, apoptosis induction [162]
	Ribozyme	Melanoma, throat carcinoma, bladder tumor	Decrease in H-ras expression; retardation of proliferation and an increase in the level of differentiation of tumor cells [ 43–47]
	siRNA	Ovary, pancreatic [163], lung [165] carcinoma	80% decrease in the protein level [165], suppression of proliferation [163,164], changes in the cell-cycle schedule, and increased number of apoptotic cells [163]
*с-myc*	asON	Leukemia [170], mammary carcinoma [171]	50%–95% decrease in c-myc expression [170, 171]; complete cell cycle arrest in the G0/G1 phase [172]
	Ribozyme	Hepatoma	1.7-fold decrease of the protein level and 1.85-fold drop in porliferative activity [173]
	siRNA	Epidermoid carcinoma, neuroblastoma [175], mammary carcinoma and lung adenocarcinoma [176]	60–92% decrease in mRNA level and 55–85% inhibition of protein synthesis [175,176]; slowing and blockage of cell division [175]
*PKC-α*	asON	Lung carcinoma	90–95% decrease in PKC-α mRNA level [55]
	Ribozyme	Glioblastoma [178], prostate carcinoma [179]	Decreases protein level by 73% and proliferative activity below 90% [178]; restores cysplatin sensitivity [179]
*Clusterin*	asON OGX-001	Renal carcinoma	Decreases clusterin mRNA level by 64% and increases cell sensitivity to paclitaxel by 80% [59]
*IGF-1R*	asON	Bladder carcinoma	Lowers mRNA level by 74% and protein level by 61.3% [207]
*bcl-2*	asON G3139 (Genasense™, USA)	Lymphoma [183], leukemia [68, 69]	Lowers bcl-2 mRNA level and Bcl-2 protein level by 60–80% and 80–95%, respectively, hence increasing cell death rate by 76–90%; as a result of apoptosis induction, increases doxorubicin senstivity [68, 69, 183]
	Ribozyme	Lymphoma	5-fold decrease in mRNA level, 3-fold decrease in protein level, and 2-fold increase in apoptosis rate [184]
	siRNA	Uteral cervix [185] and pancreatic [189] carcinoma	Suppresses Bcl-2 protein synthesis by 90%, induces apoptosis in 50% of cells [185]; increases proportion of apoptotic cells by 37% [189]
*Survivin*	asON	Malignant lung mesothelioma, glioma, mammary carcinoma, lung adenocarcinoma [76], thyroid tumor [77]	7-8- fold increase in caspase-3 activity, induction of apoptotic cell death in 42.5% of cells [76]; lowers the mRNA level by 75% and protein level by 73%, inhibits cell proliferation by 53%, 11-fold increase in the proportion of apoptotic cells [77]
	Ribozyme	Melanoma [78, 79], mammary carcinoma [80]	Lowers mRNA and protein levels 75% and 74% respectively, increases tumor cell sensitivity to chemo- and radio-therapy, no effect without an additional apoptose inducing stimulus [78 - 80]
*bcr-abl*	asON	Chronic myeloleucosis	Complete inhibition of cell growth, apoptosis induction [208]
	Maxizyme	Chronic myeloleucosis	95% decrease in the chimeric gene mRNA, apoptosis induction, tumor cell growth retardation [85]
	siRNA	Chronic myeloleucosis	Suppresses BCR-ABL-associated cell growth, 4-fold increase in tumor cell sensitivity to imanitib [87]
	DNAzyme	Chronic myeloleucosis	Suppresses protein production by 40-75% [208]
*c-raf*	asON	Lung, colon, prostate[190, 191], ovary [192, 193] cancer	100% suppression of С-raf protein, 80% inhibition of cell proliferation [190 - 192]; growth suppression in various ovary carcinoma lines by 10% to 90% [193]
	siRNA	Bladder tumor	Lowers protein level by 37.5% [194]
*MDR1*, *mdr1a/mdr1b*	asON	Colon adenocarcinoma [211], epidermoid carcinoma [212]	Complete MDR phenotype reversal, increases accumulation of doxorubicin in cells 6.4-fold, promotes cell death [211, 212]
	siRNA	Human epidermoid carcinoma [215], human pancreatic and gastric carcinoma [217], ovary cancer cells [218], murine lymphosarcoma [219]	MDR1 mRNA level is down by 91% and the P-glycoprotein by 72%-83%, increases cell sensitivity to vinblastin [99, 215], duanorubicin [217] and paclitaxel [218]
	Ribozyme	Liver cancer	Reverses MDR phenotype, increases cell sensitivity to vincristin [214]
*Glutathine*	Ribozyme	Colon cancer	Increases tumor cell sensitivity to chemotherapeutic drugs [101]
*DNMT1*	asON MG98	Lung and bladder carcinoma	Restores p16 function, promotes accumulation of hypomethylated from of retinoblastoma protein, inhibits proliferation [107]
*hTERT*	asON	Bladder cancer [111, 112]	Decreases the protein level by 97%, increases cytostatic drug sensitivty, increases proportion of apoptotic cells 3-fold, an activates caspase-3 [111, 112]
	Ribozyme	Mammary carcinoma	Reduces the length of the telomere tandem repeat region from 5.5 kbp to 3.5 kbp and reduces cell growth rate [224]
*RRR2*	asON GTI-2040 (Genasense™, USA)	Lung, bladder carcinoma, fibrosarcoma	Lowers the level of R2 subunit mRNA to below detectable levels [116]
	siRNA	Pancreatic adenocarcinoma	Increases tumor cell sensitivity to gemcytabin [198]
*VEGF*, *Flt-1* (VEGFR1), *KDR* (VEGFR2)	asON	Mammary and bladder cancer	Decreases VEGF level by 45–83% and lowers cell survival [124]
	siRNA	Ovary, uteral cervical cancer, osteosarcoma	Reduces VEGF expression by 33–53% [125]
	Ribozyme Angizyme, Sirna Ther., USA	Lung, colon and mammary carcinoma	Causes specific cleavege of RNA-substrate and effectively lowers mRNA level in cell culture [199]
	DNAzyme	Mammary carcinoma	Lowers VEGFR-2 level by 90% and decreses cell survival by 34–65% by inducing apoptosis [200]
*neu* (HER-2/erbB2)	asON	Ovary and mammary carcinoma	Additive inhibition of tumor cell proliferation in combination with doxorubicin [205]
	Ribozyme Herzyme	Ovary and mammary carcinoma	Lowers neu mRNA levels by 40–60%, thus inhibiting cell growth [131]
*eIF4E*	asON LY2275796	Non-Hodgkin lymphoma, lung bladder, colon, porstate and mammary cancer	Decreases eIF4E protein level by 80%; lowers protein levels of Bcl-2, survivin, cyclin D1, С-myc and VEGF; and induces apoptosis [136]
*PTN* and *ALK*	Ribozyme (anti-PTN)	Melanoma	Decreases PTN mRNA level by 75% [140]
	Ribozyme (anti-ALK)	Glioblastoma	Decreases PTN activity [143]
*MMP9*	Ribozyme	Prostate cancer	Causes complete degradation of MMP9 mRNA [220]
	siRNA	Juvenile osteosarcoma	50% decrease in cell migration [221]
*FGF-BP*	Ribozyme	Prostate and colon carcinoma [152, 222]	80% suppression of FGF-BP protein synthesis; slowing down of tumor cell proliferation [152, 222]
*EGR-1*	DNAzyme	Mammary carcinoma	6-fold decrease of protein level, blocking of proliferation, and 3-fold drop in tumor cell invasion activity [157]
*FAK*	siRNA	Prostate and mammary carcinoma	Inhibits tumor cell adhesion, migration, and proliferation [158]
*CXCR4*	siRNA	Mammary carcinoma	Inhibits cell migration and invasion by more than 70% [225]

## Ras

### In vitro. 

The established location of point mutations in the ras oncogene mRNA allows the development of oligonucleotides specifically targeted at the mutated sites in the mRNA target. Such drugs efficiently switch off oncogene expression. The first drug developed for ras oncogene suppression was a thiophosphate olignucleotide asON (now known as ISIS 2503) targeted at the initiation codon of Hras mRNA [161]. ISIS 2503 treatment of cervical carcinoma HeLa cells transfected with a plasmid encoding a gene for ras-luciferase fusion protein led to 98% suppression of the reporter gene. In a study by Chinese researchers, treatment of human hepatoma cells by a thiophosphate asON for 5 days caused an 87.8% inhibition of tumor cell growth [162]. Additionally, a blockage of H-ras-dependant entry of tumor cells into the S-phase of the cell cycle was observed, and DNA fragmentation detected in the treated cells indicated the initiation of apoptosis [162].

The antitumor potential of ribozymes targeted at Ras family genes is being actively investigated. In the melanoma, throat carcinoma and bladder tumor cell models, several research groups have demonstrated that H-ras ribozymes induced apoptosis, inhibited the proliferation of tumor cells, and helped reestablish cell differentiation [44-47].

An alternative approach to the suppression of Ras-family gene expression uses RNAi technology. Retrovirus-mediated expression of siRNA targeted at H-ras and K-ras mRNA effectively suppressed the synthesis of these proteins in ovarian and pancreatic carcinoma cells [163, [Bibr R164]]. The tumor proliferation activity decreased as the cells accumulated in the G0/G1 phase of the cell cycle, their proportion reaching 66.2% [163] and the number of apoptotic cells increasing from 4% to 21% [163]. Zhang and coauthors used an adenoviral system of K-ras-siRNA delivery into cells and obtained an 80% decrease in the amount of K-ras protein in lung tumor cells, along with the suppression of tumor-cell proliferation [165].

### In vivo.

The transfection of hepatocellular carcinoma cells by H-ras asON produced a decrease in the average tumor weight in mice [162, 166] and inhibited metastasis [166]. The antitumor efficiency of asON ISIS 2503 in mice with prostate tumors increased upon the addition of LNA nucleotides into the oligonucleotide sequence [167]. AsON ISIS 2503 was approved for clinical trials. In a phase I trial, the patients received 10 mg per kg asON ISIS 2503 injections for 14 days [168]. After a one week recess, the injection course was repeated. AsON ISIS 2503 did not display any marked toxicity and prevented the further progression of the disease. The results of phase II trials, performed on patients suffering from pancreatic adenocarcinoma have established that combined treatment by asON ISIS 2503 and Gemcytabine is well tolerated and demonstrated a positive response to treatment in 10.4% of patients [48].

The use of anti-H-ras ribozymes in vivo led to a significant retardation of tumor growth in mice and to a decrease in their invasive potential, as well as a two-fold increase in mouse lifespan [43-45]. Kijima and coauthors developed a ribozyme targeted at codon 12 of the mutant K-ras gene (GGT triplet substituted for GTT ) and obtained a recombinant adenovirus expressing this ribozyme [169]. An intratumor injection of this drug into athymic mice with a transplanted pancreatic carcinoma caused tumor regression in 68% of mice [169].

In vitro treatment of tumor cells with a retroviral vector containing siRNA targeted at K-ras and H-ras mRNA caused a complete inhibition of pancreatic carcinoma growth and 80% inhibition of ovary tumors in mice [163, 164]. A onetime intratumor injection of adenoviral siRNA targeted at K-ras caused a 45% inhibition of lung tumor development, and multiple injections completely stopped tumor growth in 8 out of 10 mice. In these experiments, the apoptotic activity of tumor cells showed a 2.8-fold increase [165].

## C-myc

### In vitro. 

The first drug developed for specific suppression of the c-myc protooncogene was a phosphodiester, asON, which caused a 50% decrease in the protein level in leukemia cells and inhibited their proliferation by 50% after 5-day incubation with the olignucleotide [170]. Watson et al. developed a thiophosphate asON which demonstrated a more prolonged (up to 9 days) and effective (75%) inhibition of breast cancer cells proliferation and caused a 95% inhibition of estrogene-induced overexpression of c-myc gene [171]. The next step involved the substitution of the thiophosphate backbone for a morpholino-phosphorodiamidate one. Such an anti-c-myc-asON not only caused a decrease in the protein level, but also a complete G0/G1 cell cycle blockage [172].

A «hammer head» type ribozyme was developed in order to suppress c-myc activity. Transfection by a retroviral vector encoding the ribozyme led to a 1.7-fold decrease of the protein level in hepatoma cells and a 1.85-fold decrease in the proliferation potential of the cell [173].

The ability to suppress C-myc protein hyperactivity was evaluated for gene-targeted siRNA drugs. It was shown that siRNA caused a 60-92% decrease in the mRNA level and a 55-83% decrease in C-myc protein synthesis [174, 175]. The partial abrogation of c-myc expression was associated with a 2.5-fold suppression of cell growth in human epidermoid carcinoma KB-3-1 and with a complete proliferation blockage in SK-N-MC neuroblastoma [175]. In order to improve the stability and facilitate intracellular delivery, a special poly-DNP-RNA (poly-2-O-(2,4-dinitrophenyl)-oligoribonucleotide) was designed. This drug lowered the c-myc mRNA level to 15% in mammary and lung adenocarcinoma tumor cells [176].

### In vivo.

In vivo experiments showed that AVI-4126 (AVIBioPharma, United States), a morpholino-oligonucleotide developed for inhibiting c-myc expression, causes an 80% decrease in prostate tumor growth in athymic mice [50]. This drug is currently in phase II clinical trials [52]. Phase I trials showed that AVI-4126 had no serious side effects for healthy people who received a single intravenous injection of AVI-4126 with a dosage of 90 mg [51]. Also, AVI-4126 was assessed for accumulation in the prostate and mammary tumor tissues of patients who were injected with 90 mg of the drug (the test was performed 24 hours after the injection using surgically removed tumors) [51].

The inhibition of C-myc expression by RNAi looks promising in preclinical trials. Breast cancer cells transfected with a plasmid encoding anti-c-myc-siRNA did not give rise to tumors when transplanted into mice [177]. In transgenic mice with developing lymphoma, real-time RT -PCR detected a 15%-20% decrease in c-myc mRNA in the plasma of mice treated by poly-DNP-RNA [176].

## PKC-α

### In vitro.

In order to inhibit the expression of the PKC-α gene, Dean et al. performed a thorough study of the efficiency of 20 thiophosphate asON and their 2´-O-methyl analogs [55]. The most effective oligonucleotide (known under the commercial name ISIS 3521) caused a 90-95% decrease in the level of PKC-α mRNA, but its 2´-O-methyl derivative did not affect the expression of the PKC-α gene, which indicates that RNAse H activity is required [55].

In the glioblastoma model, the inhibitory potential of an anti-PKC-α ribozyme was evaluated. A 73% decrease in the protein level after ribozyme treatment was demonstrated, and proliferative activity of the tumor cells was down by 90% as compared to 50% after treatment with a control ribozyme [178]. In another study, a PKC-α-targeted ribozyme re-established prostate carcinoma cells´ sensitivity to cisplatin-induced apoptosis, increasing its rate 2.6-3.2-fold [179].

### In vivo.

Intravenous injections of ISIS 3521 to mice with three different heterotransplants (lung carcinoma, bladder, and colon cancer) caused complete tumor growth suppression in mice at a comparatively low dosage of 0.06-0.6 mg/ kg [55]. In phase I clinical trials of asON ISIS 3521, several different treatment schedules were investigated. The main cause of toxicity was thrombocytopenia and fatigue [180]. In phase II trials with a recommended treatment schedule of 2 mg/kg/day for 3 weeks, an objective treatment response was documented for one patient with ovarian carcinoma, and 2 patients with ovarian carcinoma displayed tumor regression [181]. However, the treatment of patients with metastatic colon carcinoma did not produce any statistically significant responses [182].

## Clusterin

### In vitro.

In the inhibition of clusterin expression, thiophosphate asON affected a 64% decrease in gene expression and an 80% increase in paclitaxel sensitivity in renal carcinoma [59].

### In vivo.

2´-methoxyethyl-modified thiophosphate asON OXG-011 combined with paclitaxel caused a two-fold drop in the volume of renal carcinoma in mice [59]. Phase I of the clinical trials of asON OXG-011 at the dose of 640 mg reduced clusterin levels in human tumor tissue [60]. Currently, the therapeutic potential of asON OXG-011 combined with chemotherapy is being investigated in phase II clinical trials.

## Bcl-2

### In vitro.

Anti-mRNA drugs targeted at the antiapoptotic Bcl-2 protein emerged as undisputed leaders among the gene-targeted oligonucleotides used for cancer treatment. An 18-bp phosphodiester asON, complementary to the first 6 codons of the open reading frame of bcl-2 mRNA, was developed by Kitada and coauthors. This oligonucleotide almost completely blocked Bcl-2 protein synthesis in lymphoma cells [183]. Reed et al. compared the inhibitory effects of phosphodiester and thiophosphate oligonucleotides complementary to bcl-2 mRNA on the growth of bcl-2-expressing leukemia cells [68]. It turned out that a detectable inhibitory effect of the phosphodiester asON could be observed in about half the time needed to develop a comparable effect of the thiophosphate analog. However, the latter was more effective in suppressing the growth of tumor cells. The thiophosphate asON had the same effect as the phosphodiester asON at about a 5- to 10-time lower concentration [68]. It was shown that an 80-95% reduction in Bcl-2 protein synthesis after thiophosphate asON treatment elevates the apoptosis rate and increases doxorubicin sensitivity [69]. Luzi and coauthors [184] developed a chemically modified ribozyme targeted at bcl-2 mRNA. The lipotransfection of this drug into human Raji lymphoma cells led to a 5-fold drop in the bcl-2 mRNA level and to a 3-fold drop in the protein level, which was associated with a significant increase in the proportion of apoptotic cells [184].

Fu et al. used bcl-2 siRNA to suppress Bcl-2 synthesis by 90% in cervical tumor cells HelaB2 and BGC-823, which led to apoptosis induction [185].

### In vivo.

The decrease of the Bcl-2 protein level after treatment with G3139 asON, which was targeted at the respective gene´s mRNA, was associated with the suppression of the oncogenic potential of lymphoma cells and a complete blockage of tumor growth in mice [186]. The use of G3139 in combination with cisplatin increased the anticancer chemotherapeutic effect by 70% [187].

Recently, Morris et al. [188] reported the results of phase I clinical trials of an 18-bp thiophosphate asON G3139 (Genasense ™, United States), which were complementary to the first six codons of the bcl-2 open reading frame. Its was shown that, after seven days of daily intravenous infusions at a dose of 6.9 mg/kg, patients with non-Hodgkin lymphoma registered minor side effects, such as fatigue and a reversible increase of transglutaminases in the blood serum. Subcutaneous injection of the drug turned the cancer process onto a stable, non-progressive mode in 9 out of 21 patients with non-Hodgkin lymphoma, and improved the quality of life in 3 patients (a total objective response in 57% of patients). In September 2000, the phase III trial of asON G3139 was launched on patients with chronic lymphatic leukemia and acute myeloid leukemia [70]. The clinical trials of this drug´s combinations with various chemotherapies for melanoma and prostate carcinoma in patients resistant to hormone therapy are now in progress [71, 72]. In February 2001, in 65 oncological clinics in the United States, Canada, and Great Britain, a phase III clinical trial of asON G3139 was also launched for patients with multiple myeloma.

Tumor-bearing mice, which recieved bcl-2 siRNA treatment, displayed a retardation of liver tumor growth by 66.5% [185]. Mice with heterotransplants of pancreatic tumors registered a 56% decrease in tumor volume [189].

## Raf-1

### In vitro.

The 20-bp thiophosphate asON ISIS 5132 oligonucleotide is targeted at the 3'-nontranslated region of craf mRNA. This oligonucleotide effectively inhibited c-raf mRNA accumulation and decreased the protein level in lung carcinoma and colon and prostate tumor cells [190]. The addition of 2´-methoxyethyl modifications into the oligonucleotide asON ISIS 13650 designed for c-raf suppression did not lead to a significant increase of the inhibitory potential [191]. In ovarian carcinoma cells, ISIS 5132 and ISIS 13650 induced 100% Raf-1 suppression and an 80% drop in cell proliferation [192]. Also, anti-c-raf asON ISIS 5132 and ISIS 13650 were tested on 15 ovarian carcinoma cells lines. The proliferation suppression efficacy varied from 10% to 90%. Growth inhibition was associated with apoptosis and the accumulation of cells in the G2/M and S phases of the cell cycle [193].

The comparison of c-raf suppression efficiency of asON and siRNA, which targeted the same region of mRNA, has established that 125 nM of oligonucleotide caused a 52.4% decrease in the Raf-1 protein level, while the siRNA caused only a 37.5% drop [194]. 

### In vivo.

Oligon ucleotide ISIS 5132 demonstrated a pronounced antitumor effect in mice with ovarian carcinoma heterotransplants [192]. The results obtained in in vitro and in vivo experiments helped advance the studies of ISIS 5132 anticancer activity to clinical trials. A phase I trial demonstrated that the drug was tolerated well by patients and caused only a short period of thrombocytopenia in a few cases [195]. A low toxicity treatment schedule was devised which featured the slow intravenous infusion of the drug at a daily dose of 2mg/kg in 21-day courses with a one week break in between [195]. In the phase II clinical trial, the use of this treatment scheme for patients with prostate cancer [92], ovarian cancer [93], and colon adenocarcinoma [94] demonstrated the stabilization of the cancer process in approximately 25% of the cases.

## DNMT1

### In vitro.

The specific inhibition of the DNA-methyltransferase DNMT1 in cancer cells was achieved by the use of a 2´-O-methyl thiophosphate asON MG98. The suppression of DNMT1 by an asON results in the demethylation of the p16 gene promoter, the reestablishment of its activity, the accumulation of cells with a hypomethylated retinoblastoma gene, and the inhibition of cancer cell proliferation [107].

### In vivo.

In vivo experiments with asON MG98 demonstrated a significant growth retardation and regression of intestine carcinoma and non-small cell lung cancer heterotransplants. Successful preclinical studies insured the use of MG98 in clinical trials [196]. The phase I clinical trial was focused on evaluating the toxicity and establishing a pharmacological profile of asON MG98. Intravenous administration of the drug at a daily dose of 80 mg/m2 to patients with various solid tumors for 21 days every 4 weeks proved to be relatively safe [196]. However, no significant antitumor effect was observed. Higher doses of the drug administered according to the above-mentioned scheme were not tolerated well by patients [196, 197]. In the phase II clinical trial, an improved treatment scheme was tested on patients with metastatic renal carcinoma. The dosage of 360 mg/m2 was administered twice a week for three weeks [108]. However, this treatment schedule did not yield any positive response. The authors argued that the clinical failure was due to inappropriate choice of tumor type [108].

## RRR2

### In vitro.

The study of the asON GTI-2040 targeted at RRR 2 mRNA demonstrated a complete abrogation of RRR 2 mRNA accumulation in human lung carcinoma and significantly decreased its amount in human bladder and murine fibrosarcoma tumors [116].

Duxbury et al. studied the ability of RRR 2 siRNA to increase the gemcytabine sensitivity of human pancreatic adenocarcinoma cells implanted into mice via suppressing the RRR 2 subunit synthesis. Specific siRNA molecules targeted at RRR 2 mRNA significantly increased gemcytabineinduced cytotoxicity [198].

### In vivo.

In vivo experiments demonstrated that anti- RRR 2 siRNA, in combination with gemcytabine, yield a synergistic antitumor antimethastatic effect [198]. The inhibitory potential of GTI-2040 was evaluated in animal experiments. GTI-2040 suppressed tumor growth in all experimental models, and the maximal effect was observed in the renal carcinoma model, where a 95-98% regression in the tumor was demonstrated [116]. A study of asON GTI-2040 in phase I clinical trials yielded a recommended treatment schedule with the daily administration of 185 mg/m2/day for 21 days repeated every 4 weeks. Monotherapy according to this schedule did not cause any serious side effects [117].

## VEGF

### In vitro.

In vitro experiments demonstrate that a thiophosphate asON targeting VEGF mRNA can reduce VEGF synthesis by 45-83% in breast and bladder cancer cell lines [124].

An RNAi approach was also assessed for the efficacy of VEGF expression inhibition. Vector delivery of siRNA into tumor cells caused the prolonged suppression of VEGF synthesis by 33-53% [125].

In order to therapeutically interfere with pathological angiogenesis, as an alternative strategy it was proposed to target the VEGFR-1 and VEGFR-2 receptors, but not VEGF itself. With this in mind, ribozymes complementary to regions of Flt-1(VEGFR-1) and KDR (VEGFR-2) were designed [199]. It was shown that these ribozymes specifically cleave RNA substrates in vitro and efficiently decrease the mRNA level in cell cultures. Currently, studies of the effect of DNAzymes on VEGFR-2 function are being published. Zhang and coauthors demonstrate a 90% decrease of VEGFR-2 protein level along with a 34-65% drop in the viability of breast cancer cells due to apoptosis induction [200].

### In vivo.

Experiments in vivo demonstrated that VEGF inhibition by thiophosphate asON led to a 5-fold growth retardation of renal carcinoma in mice [201].

In order to prolong the action and improve the drug permeability into the tumor cells, VEGF- targeted modified siRNA were designed. An intratumor injection of cholesterol oligoarginine-siRNA conjugate provided a 10-fold decrease in the growth rate of colon adenocarcinoma [202], and injecting an siRNA complex with a water-soluble lipopolymer led to a 1.5-fold growth retardation of prostate adenocarcinoma [203]. In these experiments, a notable decrease in tumor vascularization and reduction of VEGF expression were also observed.

In preclinical trials, the Flt-1-targeted ribozyme exerted antitumor, antiangiogenic and antimetastatic effects in a metastatic lung cancer model [199]. Mice with grafted Lewis lung carcinoma were surgically implanted with mini pumps infusing the ribozyme at a rate of 12-100mg/kg daily. After 19 days, the mini pumps were removed and new ones installed. After treatment, a 92% tumor regression and a 70-80% reduction of lung metastasis were observed [199]. In mice with grafted intestinal carcinoma, the number of metastases after treatment with anti-Flt-1 ribozyme decreased 3-fold [199]. This ribozyme was named Angiozyme (Ribozyme Pharmaceuticals, United States) and is now being evaluated in phase I and II clinical trials in patients with various cancer types. Phase I trials were concluded in June 2001 and demonstrated good tolerance and disease stabilization in 25% of patients [204]. Phase II trials in patients with malignant forms of colon and breast cancer were aimed at evaluating Angiozyme monotherapy and combinations with traditional chemotherapeutic agents [35]. It was shown that Angiozyme decreased the levels of soluble VEGFR-1 in serum, but no significant clinical response was demonstrated. These data underscore the importance of combining genetargeted and conventional chemotherapy. Promising results were obtained in studies involving patients suffering from colon tumors and featuring the simultaneous use of Angiozyme with the Saltz therapeutic protocol (a combination of bolus 5-fluoruracil, leucovorin, and irinotecan). This approach led to disease progression in only 12.5% of the cases, as opposed to 25% in chemotherapy-only patients [35].

Interesting results were obtained in vivo with a DNAzyme complementary to KDR (VEGFR-2) mRNA. After four injections of anti-VEGFR2 DNAzyme, a 75% regression of breast cancer was observed [200]. The antiangiogenic action of the DNAzyme reduced the vascularization of the tumor tissue and therefore promoted cell death in the tumor periphery.

## neu/HER-2 (ErbB-2)

### In vitro.

An asON complementary to HER -2/neu oncogene was created and studied with respect to the antisense-mediated suppression of the gene in order to increase the efficiency of conventional chemotherapy. In combination with doxorubicin, the asON additively suppressed tumor cell proliferation [205].

The plasmid-encoded ribozyme complementary to neu mRNA efficiently cleaved the RNA substrate in vitro. The transfection of ovarian carcinoma cells by such a construct caused a 50% reduction of the gene expression and a 39-42% decrease in HER -2 protein level [131].

### In vivo.

AsON targeting neu mRNA combined with doxorubicin induced a synergistic anticancer effect [205].

The efficacy of HER-2 inhibition was assessed in vivo using injections of a recombinant adenovirus encoding a HER - 2 specific rybozyme [206]. Three days after an intra-tumor injection into subcutaneously grafted mammary cell carcinoma, a 59% decrease of HER -2/neu mRNA was observged in tumor tissue. Treating such grafted mice with five weekly injections resulted in 89% tumor regression [206]. Preliminary results of phase I trials of a HER -2/neu-targeted ribozyme called Herzyme (Ribozyme Pharmaceuticals, United States) in therapy-resistant mammary tumor patients demonstrated disease stabilization but did not yield any cases of a partial or complete positive response [132].

## Other Target Genes 

Another asON was designed to inhibit IGF-1R activity. It caused the mytocin-induced death of bladder carcinoma cells in vitro [207], and the preincubation of melanoma cells with this oligonucleotide prior to injection completely blocked tumor graft development in mice [64]. The treatment of tumor cells with asON targeted against survivin helped increase the proportion of apoptotic cells [76, 77], and the use of anti-survivin ribozymes increased the sensitivity of tumor cells to radio- and chemotherapy [79-80]. In in vitro experiments, the efficacy of drugs based on asON, siRNA, ribozymes and DNAzymes specifically for suppressing BCR-ABL was demonstrated [84, 85, 87, 208, 209]. In vivo a thiophosphate asON, complementary to BCR-ABL mRNA, proved to be most successful. The intravenous injection of this drug prolonged the survival of mice with developing leukemia [210].

To suppress the expression of the MDR1 gene and sensitize the cells to cytostatics, the respective asON [211, 212], conjugate of asON with doxorubicin [213], ribozyme [214], and siRNAs targeted at different regions of the mRNA were designed [215-218]. It was discovered that these drugs greatly increased or reestablished in vitro tumor cell sensitivity to doxorubicin [211, 213], vinblastin [215], vincristin [214], daunorubicin [217], and paclitaxel [218]. In vivo, the mice, which received subcutaneous grafts of colon cancer cells expressing the anti-MDR1 ribozyme and were later treated by doxorubicin, showed an almost complete regression of hetereotransplant growth [98]. Our experiments show that anti-mdr1b siRNA is effective in supressing mdr1b expression in the cells of highly chemotherapyresistant RLS40 lymphosarcoma [219] and, when combined with chemotherapy, specifically stimulates cell death in vitro. If cyclophosphamide and anti-mdr1b siRNA are used in combination in vivo, they cause the complete regression of RLS40 lymphosarcoma in mice and increase the efficiency of cyclophosphamide therapy more than 3-fold when compared to cyclophosphamide monotherapy [99].

In order to circumvent MDR caused by the glutathione system, a ribozyme targeted at the γ-glutamylcystein synthetase was constructed. This drug increased the sensitivity of colon tumor cells to cisplatin, doxorubicin, and etoposide 1.8-, 4.9- and 1.5-fold, respectively [101].

To overcome hTERT -mediated tumor cell immortality, specific hTERT -targeted asON were developed, such as PNA, 2´-O-methyl asON, (2-5A) asON, which has a 2´5´-oligoadenilate group [111, 112]. 2´-O-methyl asON and (2-5A) asON proved to be the most successful and caused the death of 90% of the tumor cells in vitro [111, 112]. Daily intratumoral injections of (2-5A) asON for 14 days caused a more than 50% regression of a subcutaneous grafted glioma [112]. Cells transfected by eIF4E-targeted asON decreased not only the level of the factor itself, but also that of other cancer- associated proteins, such as Bcl-2, survivin, cyclin D1, C-myc, and VEGF [136]. The intravenous infusion of antieIF4E asON to tumor-bearing mice caused a 10-fold regression of the tumor and did not have any toxic effect on the animals´ healthy tissues and organs [136].

In order to suppress anomalous PTN expression, a ribozyme- based approach was suggested. In vivo experiments showed that anti-PTN ribozymes caused a more than 65% retardation of melanoma growth in mice and inhibited angiogenesis by 70-85% [140]. Moreover, non-direct inhibition of the PTN signaling pathway by ribozymes targeted at ALK gene mRNA (which encodes a tyrosine kinase receptor for PTN ) caused a 50-95% retardation of tumor growth and a two-fold increase in the mean survival time for mice with grafted glioblastoma [143].

Suppressing metalloproteinases by ribozymes did not cause the complete cell death of tumors, but it retarded tumor growth, limited its volume increase, and suppressed angiogenesis and metastasis [147, 220, 221].

A ribozyme targeted at FGF-BP causes an 80% suppression of the proteins synthesis which inhibited cell proliferation in prostate and colon carcinomas [152, 222]. Tumors transfected by an anti-FGF-BP ribozyme and later subcutaneously grafted into mice did not show any signs of progressive development [152].

In order to suppress EGR-1, a DNAzyme was constructed. It targeted the mRNA of the transcription factor EGR-1. Intratumoral injections of this drug caused a 3-fold decrease in the tumor's size [157].

Specific siRNA was developed to suppress FAK hyper function in the cells of prostate and mammary glands. Cells transfected by this siRNA demonstrated the inhibition of cell adhesion, migration, and proliferation in vitro and tumor growth suppression in vivo in mice [158].

In vivo experiments showed that intravenous injections of anti-CXCR 4 siRNA not only decreased the CXCR4 mRNA level to 10% of the control level, but also virtually blocked metastasis into the lungs [159] and suppressed the primary tumor growth [223].

Using drugs based on nucleic acids to fight cancer is one of the most promising and rapidly developing areas of modern molecular oncology. Gene-targeted oligonucleotides make it possible to inhibit key links in the malignant growth process. These drugs can not only help regulate proliferation, apoptosis, and drug resistance, but they also affect the cell-cell interactions that promote malignant progression in the entire organism. However, it is worth noting that, although gene-directed oligonucleotides share the mechanism of action (which involves switching off the target gene), the efficiency of drugs directed at suppressing the same gene, even through the same region in the mRNA target, can vary significantly.

Some companies have already undertaken the development and marketing of anticancer drugs based on gene-targeted oligonucleotides, such as asON and ribozymes, which are well recognized as forerunners of the gene-targeted therapeutic approach. As is evident from Table 3, the genes most sensitive to asON turned out to be involved in proliferation and apoptosis blockage in tumor cells. Also, genes like Flt-1 (VEGFR) and neu (HER -2) turned out to be very susceptible to the effect of ribozymes. AsON targeted at suppressing H-ras, c-myc, PKC-α, clusterin, bcl-2, c-raf, and ribozymes, which cleave the Flt-1 and neu mRNA, are currently being evaluated in clinical trials. Certain types of gene-targeted nucleic acid drugs such as DNAzymes and siRNA have only recently started to be seen as promising therapeutic tools; however, the amount of successful clinical trials is rapidly catching up with asON and ribozymes. Drugs for curing age-related retinal degeneration based on siRNA are currently being tested in clinical trials. However, siRNA for treating cancer have not been introduced into clinical trials yet.

**Table 3:** Results of nucleic acid-based drugs testing in vivo

RNA-target	Drug	Tumor type	Effect	Trial stage
*H-ras/K-ras*	asON ISIS 2503	Hepatocellular carcinoma [162, 166]	Decreased tumor weight in mice and suppressed metastatic proccesses [162, 166]. Low toxicity, optimized therapy schedule [48, 168], positive response in combination with gemcytabine in 10% of patients; one complete response, 4 partial [48]	Phase II
	Ribozyme	Throat, pancreatic and bladder carcinoma, melanoma	Suppresses mouse tumor growth as a result of an increased proportion of apoptotic tumor cells, decreases the invasive potential of the tumor, and increases animal survival time [43–45, 169]	In vivo
	siRNA	pancreatic and ovary carcinoma [163, 164], lung cancer [165]	Inhibited tumor growth [163–165]	In vivo
*c-myc*	asON AVI-4126 (AVIBioPharma, USA)	Prostate and mammary cancer [50, 51]	Suppresses tumor growth by 80% in mice [50]. Moderate toxicity, accumulation of the drug in tumor tissue [51]	Phase II
	siRNA	Mammary carcinoma [177], lymphoma [176]	Opposes tumor progression [177]; decreases с-myc mRNA level in serum down to 15–20% [176]	In vivo
*PKC-α*	asON ISIS 3521 (AffinitakTM, USA)	Bladder carcinoma, lung and colon cancer [55], ovary cancer [181], colon carcinoma [182]	Completely suppresses tumor growth in mice at a dosage of ISIS 3521 of 0.06–0.6 mg/kg [55]. Moderate toxicity in clinical trial [180]; 3 objective responses to therapy [181]; no response to therapy [182]	Phase II
Clusterin	asON OGX-001	Renal carcinoma [59], prostate cancer [60]	Caused a 2-fold decrease in tumor volume in mice when used in combination with paclitaxel [62]. Lowered clusterin levels in the patients’ pathological tissues [60]	Phase I/II
*IGF-1R*	asON	Melanoma	Completely blocked tumor development from cells transfected with the asON [64]	Ex vivo
*bcl-2*	asON G3139 (Genasense™, USA)	Lymphomas, lympholeucosis, myeloleucosis [186, 187], melanoma [71], lymphoma [188], lympholeucosis [70], prostate carcinoma [72]	Decreased tumor volume in mice [186], additive antitumor effect of asON G3139 and cysplatin [187]. Moderate toxicity in clinical trial, stabilized the tumor process, improved life quality [188]	Phase II
	siRNA	Murine liver Н22 tumor [185], pancreatic cancer [189]	Slowed liver tumor growth in mice by 66.5% [185]; decreased pancreatic cancer heterotransplant volume by 56% [189]	In vivo
*bcr-abl*	asON	Chronic myeloleucosis	2-fold increase of mice mean survival time [210]	In vivo
*c-raf*	asON ISIS 5132 (Neopharm, USA)	Ovary [93, 192] prostate cancer [92], colon adenocarcinoma [94]	Suppressed tumor growth in mice [192]. Moderate toxicity in clinic [195], stabilization of the disease in more than 25% of cases [92, 93]	Phase II
*MDR1*, *mdr1a/mdr1b*	Ribozyme	Colon adenocarcinoma	A virtually complete regression of mouse tumors in an ex vivo experiment, in combination with doxorubicin [98]	Ex vivo
	siRNA	Murine lymphosarcoma [99, 219]	A virtually complete regression of mouse tumors in an in vivo experiment, in combination with cyclophosphamide [102]	In vivo
*DNMT1*	asON MG98	Non-small cell lung cancer, colon carcinoma, metastatic renal carcinoma and other solid tumors [108, 196, 197]	Regression of tumors in nude mice [196]. No serious side effects, no clinical response from patients [108,196, 197]	Phase II
*hTERT*	asON	Glioma	A more than 50% regression of tumor [112]	In vivo
*RRR2*	asON GTI-2040 (Genasense™, USA)	Solid tumors	Inhibited tumor growth in all the experimental models studied, maximal effect in renal carcinoma. 95–98% regression [116]. No serious side effects, optimized treatment schedule [117]	Phase II
	siRNA	Pancreatic adenocarcinoma	Synergistic siRNA and hemcytabine cytotoxicity [198]	In vivo
*VEGF*, *Flt-1* (VEGFR1), *KDR* (VEGFR2)	asON	Renal carcinoma	Fivefold tumor regression [201]	In vivo
	siRNA	Colon and prostate adenocarcinoma [202, 203]	Tenfold retardation of colon adenocarcinoma growth [202] and 1.5-fold inhibition for prostate carcinoma [203], suppresses tumor vascularization [202, 203]	In vivo
	Ribozyme Angiozyme, Sirna Ther., USA	Lung carcinoma [199], colon cancer [35, 204], mammary cancer [35]	Growth regression of Lewis lung carcinoma in mice by 92% and 70–80% decrease in metastasis in the lungs [199]. Well-tolerated by patients, lowers VEGFR-1 protein level in tumor cells, stabilizes the disease in 25% of patients [35, 204]	Phase II
	DNAzyme	Mammary carcinoma	Causes tumor regression by 75% in mice, reduces vasularization in tumors, and causes cell death in the tumor’s peripherial tissue [200]	In vivo
*neu* (HER-2/erbB2)	asON	Ovary and mammary cancer	Synergistic anticancer effect with doxyrubicin [205]	In vivo
	Ribozyme Херзим	Mammary carcinoma	Regression of tumors in mice by 89% [206]. Well-tolerated by patients, stabilzes the disease [132]	Phase I
*eIF4E*	asON LY2275796	Prostate carcinoma	Tenfold regression of the tumor; has no toxic effect on healthy organs or tissues in mice [136]	
*PTN* and *ALK*	Ribozyme (anti-PTN)	Melanoma	Reduces tumor size by 65%, inhibits vascularization by 70–85%, and induces apoptosis [140]	In vivo
	Ribozyme (anti-ALK)	Glioblastoma	Slows tumor growth, increases tumor-bearing mice survival time 2-fold [143]	In vivo
*MMP9*	Ribozyme	Metastatic fibrobalsts [220], prostate cancer[147]	Causes an 8-fold decrease in metastasis, a 1/3 increase in mean mouse survival time, and has no effect on primary tumor development [147, 220]	Ex vivo
*FGF-BP*	Ribozyme	Prostate carcinoma	Suppresses tumor development [152]	Ex vivo
*EGR-1*	DNAzyme	Mammary carcinoma	Suppresses tumor growth 3-fold [157]	In vivo
*FAK*	siRNA	Prostate and mammary carcinoma	Tumor regression [158]	In vivo
*CXCR4*	siRNA	Mammary carcinoma	Virtually completely inhibits metastasis, lowers CXCR4 mRNA level to 10% [159], and causes tumor regression [223]	In vivo

Despite the undoubted achievements of modern oncology, the problem of raising the efficiency of malignant neoplasia treatment is still very pressing. Notably, one of the main objectives of gene-targeted therapy is to provide the delivery of drugs to the target cells, which presumes the transport into a specific organ or tissue, traversing the cellular membrane, and arriving at specific cell compartments. It is clear that oligonucleotides cannot do this by themselves. Therefore, the development of vehicle systems which could increase the efficiency of these drugs is of utmost importance. The problem of transporting a specific RNA or DNA sequence into a tumor cell is still unsolved.

As was noted above, changes in the expression of certain genes play a key role in oncogenesis. The dysfunction of these genes can affect the correct regulation of the signaling pathways that control transitions between the phases of the cell cycle, proliferation, apoptosis, genetic stability, differentiation, and morphogenetic reactions of the cell. These processes in the cell are tightly connected and are often interchangeable. In many cases, drugs based on nucleic acids affect a specific mechanism, which leads to the abrogation of a whole chain of malignant growth regulation. However, it is important to bear in mind that one of the most crucial properties of tumor cells is the ability to exploit cell survival pathways, which allows these cells to escape genetargeted molecular control. This consideration, however, does not undermine the importance of cancer therapy using nucleic acid-based drugs directed at a specific regulatory link. Nevertheless, the flexibility and plasticity of the biochemical profile, as well as the robustness of the regulation of functions vital for the survival of tumor cells, require that gene-targeted cancer therapy be optimized as much as possible.
